# Haphazard Intentional Sampling in Survey and Allocation Studies on COVID-19 Prevalence and Vaccine Efficacy †

**DOI:** 10.3390/e24020225

**Published:** 2022-01-31

**Authors:** Miguel G. R. Miguel, Rafael P. Waissman, Marcelo S. Lauretto, Julio M. Stern

**Affiliations:** 1 Institute of Mathematics and Statistics, University of São Paulo, São Paulo 05508-900, SP, Brazil; mgabriel@ime.usp.br; 2 School of Arts, Sciences and Humanities, University of São Paulo, São Paulo 03828-000, SP, Brazil; rafaelwaissman@usp.br (R.P.W.); marcelolauretto@usp.br (M.S.L.)

**Keywords:** haphazard intentional sampling, rerandomization, pure randomization, optimal sampling design

## Abstract

Haphazard intentional sampling is a method developed by our research group for two main purposes: (i) sampling design, where the interest is to select small samples that accurately represent the general population regarding a set of covariates of interest; or (ii) experimental design, where the interest is to assemble treatment groups that are similar to each other regarding a set of covariates of interest. Rerandomization is a similar method proposed by K. Morgan and D. Rubin. Both methods intentionally select good samples but, in slightly different ways, also introduce some noise in the selection procedure aiming to obtain a decoupling effect that avoids systematic bias or other confounding effects. This paper compares the performance of the aforementioned methods and the standard randomization method in two benchmark problems concerning SARS-CoV-2 prevalence and vaccine efficacy. Numerical simulation studies show that haphazard intentional sampling can either reduce operating costs in up to 80% to achieve the same estimation errors yielded by the standard randomization method or, the other way around, reduce estimation errors in up to 80% using the same sample sizes.

## 1. Introduction

Large-scale sampling and experimental design problems usually demand large staff and infrastructure and expensive field operations to cover a representative group of the population of interest. Even then, pure (or stratified) randomized experiments do not guarantee efficient control over specific sets of covariates, and there may be large divergences between sample and population statistics. To address this problem, Lauretto et al. [1,2] and Fossaluza et al. [3] developed the haphazard intentional sampling method, an approach that combines intentional sampling, using methods of numerical optimization for an appropriate objective function, with random disturbances ensuring good decoupling properties. The word ‘haphazard’ was used by Dennis Lindley to distinguish the decoupling effect from the tool used to obtain the desired decoupling, namely, randomization; for further details, see [1,3,4]. For a fixed sample size, this technique aims at diminishing the distance between sample and population regarding specific covariates of interest or, the other way around, minimizing the sample size needed to achieve good enough expected agreement between sample and population regarding specific covariates of interest. The Mahalanobis distance is the natural choice for the statistical model at hand, but other $L_{p}$ distances, or convex combinations thereof, will be used as approximations useful for numerical computation, as explained in the following sections. This method can be applied in several contexts, such as allocations of treatment and control groups in medical trials [2] or in statistical sampling problems [5]. In this method, a weight factor, λ, adjusts the weight of the random perturbation relative to the deterministic objective function of the optimization problem. In practical problems, the weight factor λ can be calibrated in such a way that, on the one hand, it is small enough to generate only slightly sub-optimal solutions and, on the other hand, it is large enough to break potential confounding effects that could introduce spurious statistical biases in the study.

In this paper, the performance of the haphazard intentional sampling method is compared to pure random sampling and to the rerandomization methods proposed by Morgan and Rubin [6]. As benchmarks, we use two case studies. The first case study, presented in Section 2, concerns the prevalence of SARS-CoV-2, using covariates from public data sets generated by the 2010 Census of the Brazilian Institute of Geography and Statistics (IBGE). The second case study, presented in Section 3, concerns a multiple group allocation problem where the interest is to compare the efficacy of four different COVID-19 vaccines. The performance of the aforementioned methods is compared in our case studies in a batch of numerical simulations regarding the proximity of generated samples to covariate means of the total population and the precision of ensuing statistical estimators.

## 2. Haphazard Intentional Sampling Method: Two-Group Allocation

In this section, we present the formulation of the haphazard intentional sampling method presented by Lauretto et al. [1,2]. Let X denote a matrix in $R^{n\times d}$, where *n* is the number of candidate sampling units and *d* is the number of covariates of interest. A two-group allocation consists of assigning to each unit a group chosen from a set of possible groups, G={0,1}, where 0 and 1 usually denote the control and treatment groups, or the unsampled and sampled units. We denote by w an allocation vector in $G^{n}$, assigning each unit to a group. We also assume that the number of units assigned to each group is previously defined. That is, integers $n_{1}$ and $n_{0}$ exist such that $n_{1}+n_{0}=n$, $1w^{t}=n_{1}$ and $1{\left(1-w\right)}^{t}=n_{0}$. 1 denotes a vector of ones with the proper size; therefore, the scalar product $1w^{t}$ is the sum of the scalar components of w. The goal of the allocation problem is to generate an allocation, w, that, with high probability, approximately minimizes the imbalance between groups with respect to a loss function, L(w,X).

The Mahalanobis distance is the metric of choice for statistical models based on the multivariate normal distribution; for further details, see (Stern [7], Section 6.2). The Mahalanobis distance between the covariates of interest in each group is defined as follows. Let A be an arbitrary matrix in $R^{n\times m}$. Furthermore, define $A^{*}:=AL$, where L is the lower triangular Cholesky factor [8] of the inverse of covariance matrix of A; that is, $\mathrm{Cov}{\left(A\right)}^{-1}=LL^{t}$.

For an allocation w, let ${\bar{A^{*}}}^{1}$ and ${\bar{A^{*}}}^{0}$ denote the averages of each column of $A^{*}$ over units allocated to, respectively, groups 1 and 0 according to the row vector w:

(1) $${\bar{A^{*}}}^{1}:=\left(1/n_{1}\right)wA^{*}\;\;\mathrm{and}\;\;{\bar{A^{*}}}^{0}:=\left(1/n_{0}\right)\left(1-w\right)A^{*}\;.$$

The Mahalanobis distance between the average of the column values of A in each group specified by w is defined as:

(2) $$M\left(w,A\right):=m^{-1}{∥{\bar{A^{*}}}^{1}-{\bar{A^{*}}}^{0}∥}_{2}\;\;,$$

where *m* denotes the number of columns of A.

### 2.1. Pure Intentional Sampling Formulation

Under the Mahalanobis loss function, a pure intentional sampling procedure consists of generating an allocation w that minimizes the following optimization problem:

(3) $$\begin{array}{cc} \mathrm{minimize} & M(w,X)\\ \mathrm{subject}\;\mathrm{to} & 1w^{t}=n_{1}\\ \; & 1{\left(1-w\right)}^{t}=n_{0}\\ \; & w\in {\left\{0,1\right\}}^{n} \end{array}$$

The formulation presented in Equation (3) is a Mixed-Integer Quadratic Programming Problem (MIQP) [9] that can be computationally very expensive. The hybrid loss function, H(w,A), is a surrogate function for M(w,A) built using a linear combination of $L_{1}$ and $L_{\infty }$ norms; see Ward and Wendell [10]:

(4) $$H\left(w,A\right):=m^{-1}\left(∥{\bar{A^{*}}}^{1}-{\bar{A^{*}}}^{0}{∥}_{1}+\sqrt{m}\;\;{∥{\bar{A^{*}}}^{1}-{\bar{A^{*}}}^{0}∥}_{\infty }\right)$$

The minimization of H(w,A) yields the Mixed-Integer Linear Programming Problem (MILP) defined in the next equation, which is computationally much less expensive than the MIQP problem (3); see Murtagh [11] and Wolsey and Nemhauser [9].

(5) $$\begin{array}{cc} \mathrm{minimize} & \;H(w,X)\\ \mathrm{subject}\;\mathrm{to} & 1w^{t}=n_{1}\\ \; & 1{\left(1-w\right)}^{t}=n_{0}\\ \; & w\in {\left\{0,1\right\}}^{n} \end{array}$$

Statistical inference based on pure intentional sampling is vulnerable to malicious manipulation, unconscious biases, and many other confounding effects. In the Frequentist School of statistics, the use of intentional allocation is anathema, whereas in the Bayesian School, it has been the subject of long-standing debates. The solution presented in this paper is a compromise aiming to achieve the effective performance of intentional sampling but using moderate randomization to avoid systematic confounding effects. Lauretto et al. [1] and Fossaluza et al. [3] provide a thorough discussion of the motivation and history of the ideas leading to the haphazard intentional sampling method.

### 2.2. Haphazard Formulation

The haphazard intentional sampling method consists of extending the pure intentional sampling method, formulated in Equation (5) as a MILP optimization problem, with the introduction of a noisy component. Let Z be an artificially generated random matrix in $R^{n\times k}$, with elements that are independent and identically distributed according to the standard normal distribution. For a given tuning parameter, λ∈[0,1], the haphazard method aims to solve the following optimization problem:

(6) $$\begin{array}{cc} \mathrm{minimize} & \left(1-\lambda )\;H(w,X)+\lambda \;H(w,Z\right)\\ \mathrm{subject}\;\mathrm{to} & 1w^{t}=n_{1}\\ \; & 1{\left(1-w\right)}^{t}=n_{0}\\ \; & w\in {\left\{0,1\right\}}^{n} \end{array}$$

The parameter λ controls the amount of perturbation that is added to the surrogate loss function, H(w,X). If λ=0, then $w^{*}$ is the deterministic optimal solution for H(w,X), corresponding to the pure intentional sampling. If λ=1, then $w^{*}$ is the optimal solution for the artificial random loss, H(w,Z), corresponding to a completely random allocation. By choosing an intermediate value of λ (as discussed in Section 2.3.2), one can obtain $w^{*}$ to be a partially randomized allocation such that, with a high probability, $H(w^{*},X)$ is close to the minimum loss.

### 2.3. Case Study: Estimating SARS-CoV-2 Infection Prevalence

The artificial data set used for the simulations carried in this study is inspired by the Epicovid19 Project [12], a survey conducted by the Brazilian Institute of Public Opinion and Statistics (IBOPE) and the Federal University of Pelotas (UFPel) to estimate SARS-CoV-2 infection prevalence in 133 Brazilian municipalities. Our study is supplemented by data from the 2010 Brazilian census conducted by IBGE, giving socio-economic information by census sector. Sectors are the minimal units by which census information is made publicly available. Typically, each sector contains around 200 households. Furthermore, households in a sector form a contiguous geographic area with approximately homogeneous characteristics.

The first step of the sampling procedure of Epicovid19 study consisted of randomly selecting a subset of census sectors of each surveyed municipality. As a second step, at each of the selected sectors, a subset of households was randomly selected for a detailed interview concerning socio-economic characteristics and SARS-CoV-2 antibody testing. Our benchmark problem is based on the original Epicovid19 study, where we evaluated the impact of alternative census sector sampling procedures on the estimation of the response variable, namely, SARS-CoV-2 prevalence. In order to simulate outcomes for alternative sector selections, we used an auxiliary regression model for this response variable, as explained in the sequel.

#### 2.3.1. Auxiliary Regression Model for SARS-CoV-2 Prevalence

The auxiliary regression model for SARS-CoV-2 prevalence had the Epicovid19 estimated infection rates adjusted for the spread of the pandemic in subsequent months and corrected for under-reporting due to lack of intensive testing in Brazil. As explanatory variables, this auxiliary model used 15 socio-demographic covariates, including income, ethnicity, age, sanitation condition, etc. The parameters of this auxiliary regression model were estimated using standard regression packages available in the R statistical environment. Since the response variable is simulated by this auxiliary regression model, its covariates and their weight coefficients in the regression can be taken as a valid representativity target, that is, the haphazard and rerandomization methods will try to make sector selections that resemble the population characteristics corresponding to these 15 covariates.

The auxiliary model was a logit link regression, specified by selecting, via a stepwise procedure ([13], Section 15.2), three of the most relevant predictive variables, namely, average income, population percentage with zero income, and percentage of households with two or more bathrooms (a standard indirect measure of wealth used by IBGE):

(7) $$ln\left(p_{i}/\left(1-p_{i}\right)\right)=\eta_{i}=\hat{\beta_{0}}+\hat{\beta_{1}}x_{i,1}+\hat{\beta_{2}}x_{i,2}+\hat{\beta_{3}}x_{i,3}+\epsilon_{i}$$

(8) $$p_{i}=\frac{e^{\eta_{i}}}{\left(1+e^{\eta_{i}}\right)}$$

$p_{i}$: simulated SARS-CoV-2 prevalence in sector *i*;
$x_{i,1}$: income in census sector *i*;
$x_{i,2}$: zero-income population percentage in census sector *i*;
$x_{i,3}$: percentage of households with two or more bathrooms in census sector *i*.

#### 2.3.2. Balance and Decoupling Trade-Off in the Haphazard Method

The haphazard intentional sampling method is not exclusively concerned with choosing maximally representative samples. Equally important is to prevent estimation biases induced by spurious confounding effects. This is exactly the role of the decoupling effects engendered by standard randomization procedures. We need a quantitative measure to assess how effectively the noise introduced in the method, with weight λ, is performing this task. A proxy measure of this sort can be constructed using the Fleiss Kappa coefficient, conceived to measure the degree of agreement between nominal scales assigned by multiple raters, see Fleiss [14]. In our context, it is used as follows.

For *r* repetitions of a sampling procedure, let $r_{i,j}$ denote the number of times element i∈{1,2,…,N} is allocated to group j∈{0,1}. Let ${\bar{P}}_{o}$ denote the observed average proportion of concordance among all allocation pairs. Let ${\bar{P}}_{e}$ denote the expected agreement that would be obtained by chance, conditional on the proportion of assignments that were observed in each group *j*.

(9) $${\bar{P}}_{o}=\frac{1}{Nr(r-1)}\sum_{i=1}^{N}\sum_{j=0}^{1}r_{i,j}\left(r_{i,j}-1\right)\;{\bar{P}}_{e}=\sum_{j=0}^{1}\frac{{\left(\sum_{i=1}^{N}r_{i,j}\right)}^{2}}{{\left(Nr\right)}^{2}}$$

The Fleiss Kappa coefficient is obtained by the ratio of the difference between the observed and the expected random agreement, ${\bar{P}}_{o}-{\bar{P}}_{e}$, and the difference between total agreement and the agreement obtained by chance, $1-{\bar{P}}_{e}$:

(10) $$\kappa =\frac{{\bar{P}}_{o}-{\bar{P}}_{e}}{1-{\bar{P}}_{e}}$$

The relationship between decoupling and the degree of disturbance added is assessed empirically. The following transformation between parameters λ and $\lambda^{*}$ is devised to equilibrate the weights given to the terms of Equation (6) corresponding to the covariates of interest and artificial noise, according to dimensions *d* (the number of columns of X) and *k* (the number of columns of Z).

(11) $$\lambda =\lambda^{*}\;/\;\left[\lambda^{*}\left(1-k/d\right)+k/d\right]\;,\;\;\mathrm{where}\;\lambda^{*}\in \left\{0.005,0.01,0.05,0.1,0.25,0.5\right\}.$$

**Figure 1:**
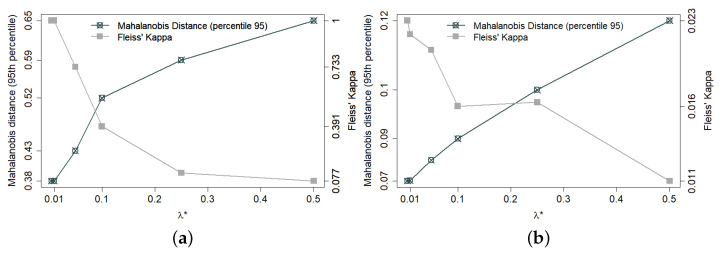
Trade-off between balance and decoupling in 300 allocations for two municipalities containing, respectively, 34 (**a**) and 176 (**b**) sectors. Sectors are the minimal units by which census information is made publicly available, each containing about 200 households. Balance between allocated and non-allocated sectors is expressed by the 95th percentile of Mahalanobis distance. Decoupling is expressed by Fleiss’s Kappa coefficient—notice the different range in each case (**a**,**b**).

The trade-off between balancing and decoupling also varies according to the characteristics of each municipality. Small municipalities have only a limited number of census sectors and, hence, also a limited set of near-optimal solutions. Therefore, for small municipalities, good decoupling requires a larger $\lambda^{*}$. Figure 1a shows, for the smallest of the 133 municipalities in the database (with 34 census sectors), the trade-off between balance and decoupling (Fleiss’s Kappa) as $\lambda^{*}$ varies in proper range. Figure 1b shows the same trade-off for a medium-size municipality. Since it has many more sectors (176), it is a lot easier to find well-balanced solutions and, hence, good decoupling is a lot easier to achieve.

Larger municipalities engender larger optimization problems (for the number of binary decision variables equals the number of census sectors) that, in turn, usually require more CPU time for the MILP solver. Table 1 displays empirically calibrated parameters $\lambda^{*}$ and maximum CPU times under the hardware configuration described in Section 2.3.3.

#### 2.3.3. Benchmark Experiments and Computational Setups

Our performance experiments used a subset of 10 municipalities of the 133 in the original Epicovid19 study, covering a wide range of population size and characteristics. Following the original Epicovid19 protocol, a sample of 25 census sectors was selected at each municipality. The sampling procedure for selecting these 25 sectors was repeated 300 times, using each of the three methods under comparison, namely, haphazard method, rerandomization, and pure randomization.

Numerical optimization and statistical computing tasks were implemented using the R v.3.6.1. environment [15] and the Gurobi v.9.0.1 optimization solvers [16]. The computer used to run these routines had an AMD RYZEN 1920X processor (3.5 GHz, 12 cores, 24 threads), ASROCK x399 motherboard, 64 GB DDR4 RAM, and Linux Ubuntu 18.04.5 LTS operating system. There is nothing specific about hardware configuration, with performance being roughly proportional to general computing power.

### 2.4. Experimental Results

In this section, we present the comparative results for the haphazard, rerandomization, and simple randomization methods, considering the metrics discussed in the sequel.

#### 2.4.1. Group Unbalance among Covariates

We compute the standardized difference between group means for each covariate, based on 300 simulated allocations per method. Specifically, we compute the empirical distribution of the statistics $\left({\bar{X}}_{•,j}^{1}-{\bar{X}}_{•,j}^{0}\right)/s_{j}$, where ${\bar{X}}_{•,j}^{1}$ and ${\bar{X}}_{•,j}^{0}$ denote the averages of the *j*-th column of X over units allocated to, respectively, groups 1 and 0 (see Equation (1)); and $s_{j}$ is the reference scale given by the standard deviation of ${\bar{X}}_{•,j}^{1}-{\bar{X}}_{•,j}^{0}$ computed over 300 pure random allocations.

**Figure 2:**
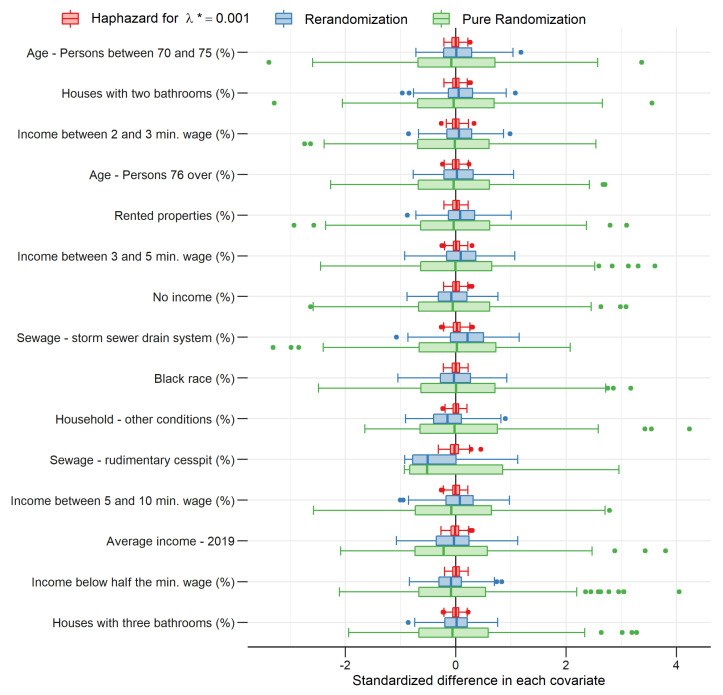
Difference between groups 1 (sampled sectors) and 0 (not sampled sectors) with respect to average standardized covariate values for each type of allocation.

Figure 2 shows the distribution of standardized differences in each covariate (see Morgan and Rubin [17]) for São Paulo, the largest Brazilian municipality (18,182 sectors). It can be easily seen that differences are remarkably smaller for the haphazard allocations than for the rerandomization allocations, which, in turn, are remarkably smaller than for the pure randomization allocations. It is important to mention that this same pattern is verified in all other municipalities.

#### 2.4.2. Root Mean Square Errors of Simulated Estimations

We now consider simulated scenarios where, once we have sampled the sectors in each municipality, we estimate the municipality’s SARS-CoV-2 prevalence based on observed prevalences on these sectors. Here, SARS-CoV-2 prevalence in each sector is simulated by the auxiliary regression model described in Section 2.3.1.

To assess the estimation error and variability yielded in each sampling method, we compute, for each municipality, the root mean square error (RMSE) and the standard deviation (SD) of estimates, as follows:

(12) $$RMSE\left(\hat{\theta }\right)=\sqrt{\frac{1}{r}\sum_{a=1}^{r}{\left({\hat{\theta }}_{a}-\theta \right)}^{2}}\;SD\left(\hat{\theta }\right)=\sqrt{\frac{1}{r-1}\sum_{a=1}^{r}{\left({\hat{\theta }}_{a}-E\left(\hat{\theta }\right)\right)}^{2}}\;,$$

where r=300 denotes the number of allocations, ${\hat{\theta }}_{a}$ denotes the SARS-CoV-2 prevalence estimated from allocation *a*, θ denotes the SARS-CoV-2 prevalence considering all sectors of the municipality, and $E(\hat{\theta })$ denotes the average of ${\hat{\theta }}_{a}$ computed over *r* allocations.

Table 2 presents the $RMSE(\hat{\theta })$ and $SD(\hat{\theta })$ yielded by each sampling method for the 10 municipalities selected for this study. Both the haphazard and the rerandomization methods show RMSEs and SDs that are much smaller than the pure randomization method. Moreover, the haphazard method outperforms the rerandomization method, in the following sense: (a) The haphazard method yields smaller estimation errors (measured by RMSE) than the rerandomization methods (in 9 out of 10 municipalities for this simulation); (b) moreover, estimation variability (measured by SD) is substantially smaller for the haphazard method in all municipalities.

**Figure 3:**
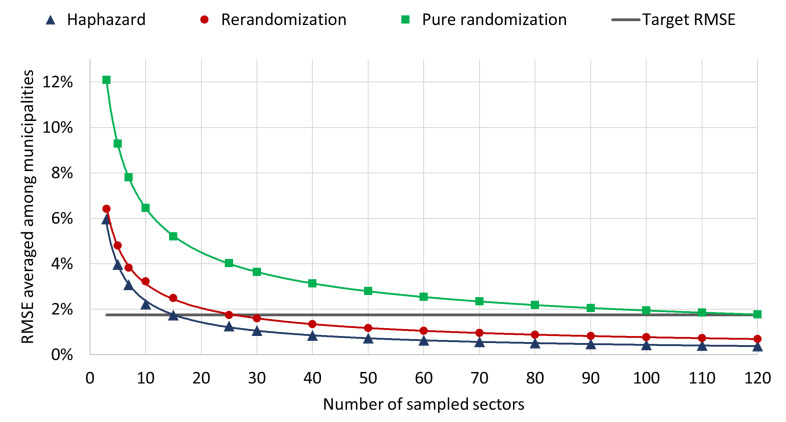
RMSE averaged among municipalities × number of sampled sectors.

The RMSEs analyzed in the last paragraphs can be used to compute the sample size required to achieve a target precision in the statistical estimation of SARS-CoV-2 prevalence. As mentioned in Section 2.3, each sampling unit consists of a census sector containing around 200 households; the sample size refers to the number of sectors to be selected from each municipality. Figure 3 shows RMSEs as a function of sample size. If the sample size for each municipality is calibrated in order to achieve the target precision of the original Epicovid19 study (black horizontal line), using the haphazard method implies an operating cost 40% lower than using the rerandomization method and 80% lower than using pure randomization.

## 3. Multiple-Group Allocation

This section introduces an explicit notation for the haphazard intentional sampling method for a case with multiple groups. A naive treatment for multiple groups extending our previous formulation for two groups would be to compute all pairwise comparisons between groups, using the Mahalanobis distance, as first suggested in Lock [18]. It can be shown that minimizing the sum of squared Mahalanobis distances between all group pairs is equivalent to minimizing the sum of squared Mahalanobis distances between each group and the overal mean, see (Blum, Hopcroft and Kannan [19], Lemma 7.1, p. 186). More specifically, we can minimize the Mahalanobis distance between the mean of each group and the overall mean of the entire dataset.

Let $W\in {\left\{0,1\right\}}^{g\times n}$ be an allocation matrix, where *g* is the number of groups, *n* is the number of candidate sampling units and $W_{q,i}=1$ denotes the assignment of element *i* to group *q*. The number of experimental units in each group is given by the vector $n=\{n_{1},n_{2}\cdots n_{g}\}$. Each row of W refers to an allocation vector which, as in Equation (1), is associated with a subset of the overall data matrix, A, so that the vector of covariate averages in group *q* is $\left(1/n_{q}\right)W_{q,•}A$. As already explained in Section 2, the Mahalanobis distance is computed on vector averages normalized and rotated by the Cholesky factor of the inverse of covariance matrix of A, namely,

(13) $${\bar{A^{*}}}^{q}:=\left(1/n_{q}\right)W_{q,•}A^{*}.$$

The well-known k-means algorithm is based on the minimization of the sum of squared distances between groups. Analogously, we set the naive goal of obtaining an optimal allocation by minimizing the summation over pairwise squared Mahalanobis distances between groups, namely:

(14) $$M_{pairs}^{2}\left(W,A\right):=m^{-1}\sum_{1\leq q_{1}<q_{2}\leq g}{∥{\bar{A^{*}}}^{q_{1}}-{\bar{A^{*}}}^{q_{2}}∥}_{2}^{2}\;\;.$$

As already mentioned in the first paragraph of this section, we can replace the minimization over the sum of pairwise squared distances by the minimization of the sum of squared distances between each group’s average to a central mean (Blum, Hopcroft and Kannan [19], Lemma 7.1, p. 186), namely,

(15) $$M_{centroid}^{2}\left(W,A\right):=m^{-1}\sum_{q=1}^{g}{∥{\bar{A^{*}}}^{q}-\bar{A^{*}}∥}_{2}^{2}\;\;,$$

where $\bar{A^{*}}:=\left(1/n\right)1A^{*}$.

In the context of statistical sampling, the last equation can be interpreted as obtaining groups that are good representatives of the entire population. In the last section, we used the hybrid heuristic loss function as a proxy for the Mahalanobis distance in order to replace the implied quadratic minimization problem by a much easier to solve linear optimization problem. In the same way, in this section we replace the last quadratic optimization problem by the following minimization of sum of Hybrid loss functions:

(16) $$H_{centroid}\left(W,A\right):=m^{-1}\sum_{q=1}^{g}\left(∥{\bar{A^{*}}}^{q}-\bar{A^{*}}{∥}_{1}+\sqrt{m}\;\;{∥{\bar{A^{*}}}^{q}-\bar{A^{*}}∥}_{\infty }\right)\;\;.$$

Hence, this formulation of the haphazard method for multiple groups is rendered by the following MILP:

(17) $$\begin{array}{cc} \mathrm{minimize} & \left(1-\lambda \right)\;H_{centroid}\left(W,X\right)+\lambda \;H_{centroid}\left(W,Z\right)\\ \mathrm{subject}\;\mathrm{to} & W\;1=n\\ \; & 1\;W=1\\ \; & W\in {\left\{0,1\right\}}^{g\times n} \end{array}$$

As in Section 2, parameter λ∈[0,1] defines the amount of noise in the allocation process, which has to be calibrated for each application problem.

### 3.1. Case Study: Vaccine Efficacy Testing

With the emergence of COVID-19 pandemic, the Butantan Institute, a Brazilian biological research center, signed in 2020 an agreement with the pharmaceutical company Sinovac for the import and production of Coronavac vaccine in Brazil [20]. As part of the efforts to assess the effectiveness of Coronavac, the Butantan Institute carried out an epidemiological study in Serrana, a Brazilian medium-size municipality, in which the entire adult population received the recommended doses of vaccine and was monitored for infections, hospitalizations and deaths. That epidemiological study, carried out in July 2020–January 2021, was called *S Project* [21].

In January 2021, alternative vaccines became available for potential mass use in Brazil, motivating the case study presented in this section, that simulates the use of four different COVID-19 vaccines. The use of the haphazard intentional sampling and the rerandomization methods, and their effect in multigroup allocation optimization, are clearly demonstrated in this simulated study, motivating the use of these methods in the design of future trials and statistical studies concerning vaccine efficacy.

Efficacy refers to the degree to which a vaccine prevents symptomatic infection under controlled circumstances such as clinical trials. Specifically, the vaccine’s efficacy is measured by the ratio between sickness rate in volunteers who got the vaccine and sickness rate in volunteers who got the placebo [22,23]. Main vaccines had their Phase III clinical trials conducted in similar time frames, between the third quarter of 2020 and the first quarter 2021 ([23], Table 2).

The allocation dataset of this simulated study included all 45 census sectors in the municipality of Serrana, considering the same 15 socio-demographic covariates as the case study presented in Section 2.3. For operational reasons commonly adopted in this kind of study, we consider the scenario in which the population of each census sector receives the same vaccine and the 45 census sectors are allocated in four groups, of sizes (12, 11, 11, 11), using the three methods under study. Infections before the administration of any vaccine are simulated according to the Gaussian linear model described in Section 2.3.1. Hence, the expected infection rate before vaccination is given by $p_{i}$, the SARS-CoV-2 prevalence in sector *i*, see Equations (7) and (8). The infection rates after the administration of a given vaccine are reduced according to the expression $IR_{v,i}=p_{i}\left(1-ER_{v}\right)$, where $ER_{v}$ is the efficacy rate of vaccine *v* published by each vaccine’s manufacturer, as shown in Table 3.

### 3.2. Experimental Results

**Figure 4:**
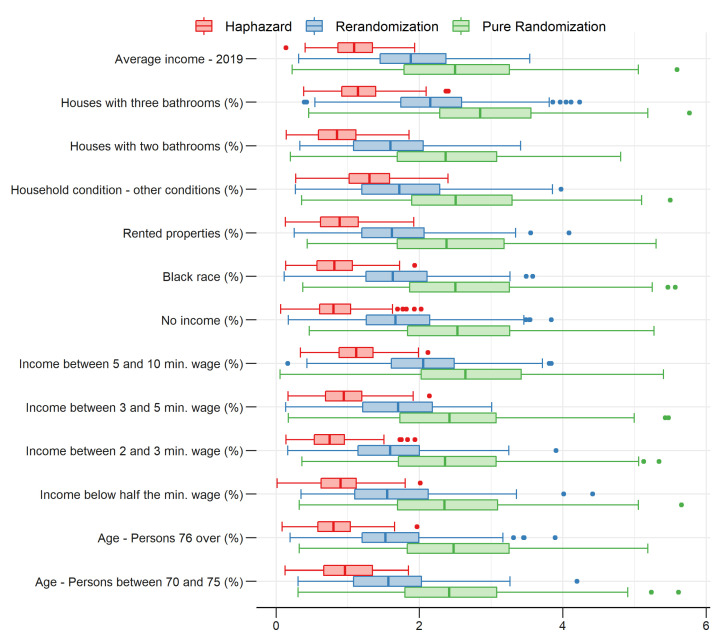
Maximum pairwise standardized differences in each covariate.

We simulate and compute the standardized difference between group means for each covariate, based on 300 simulated allocations per method, using the same procedure described in Section 2.3. Figure 4 displays boxplots of maximum pairwise standardized differences in each covariate. More specifically, the boxplots compare a measure of unbalance among groups in each covariate *j*, defined as $\max_{q_{1},q_{2}}\left|{\bar{X}}_{•,j}^{\left(q_{1}\right)}-{\bar{X}}_{•,j}^{\left(q_{2}\right)}\right|/s_{j}$ for simulated allocations obtained for each method, where indices $q_{1},q_{2}\in \left\{1\ldots 4\right\}$ and $s_{j}$ is a standard deviation defined as in Section 2.4.1. As clearly seen, the haphazard intentional sampling method outperforms the rerandomization method that, in turn, outperforms the pure randomization method. The difference in performance between these methods in this four group allocation case study is even greater than in the first case study concerning a two group allocation.

**Figure 5:**
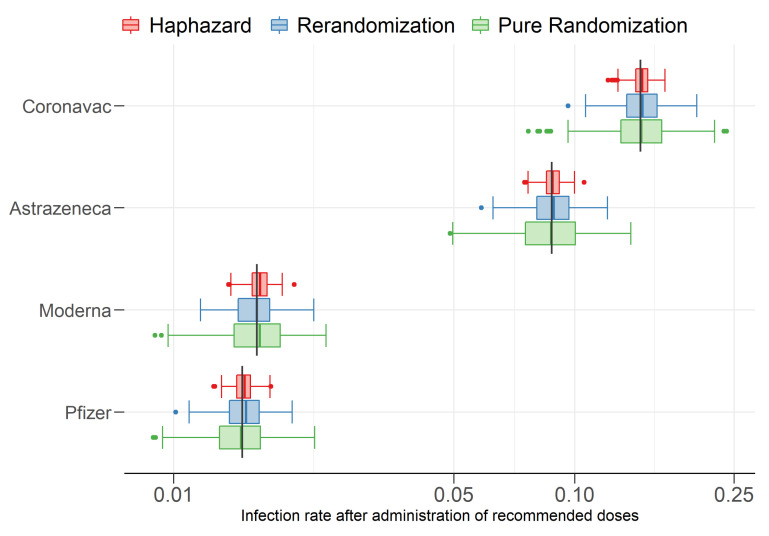
Estimated (boxplots) and actual (vertical lines) COVID-19 infection rates after administration of recommended doses for each vaccine.

Figure 5 displays boxplots for the corresponding predictive quantity of interest, namely, the infection rates after the administration of each vaccine. The difference in performance between the methods is exactly as expected from the difference in performance in balancing covariates. Table 4 displays the RMSE and corresponding standard deviation (SD) for the infection rates in the 300 simulations. Overall, the RMSE of the haphazard method was, respectively, 2 and 3 times lower than those of rerandomization and pure randomization methods.

## 4. Discussion

Both the haphazard and rerandomization methods proved to be reliable and robust, outperforming the standard randomization method. Moreover, the haphazard method consistently outperformed the rerandomization method. This increased performance has a direct impact in the design and implementation of clinical trials allowing a target precision of experiment to be achieved with a reduced sample size. Reduced sample sizes immediately imply reduced costs of implementation as well as a faster conclusion of the experiment. Even more, reduced sample sizes help to mitigate ethical concerns related to potential side effects and other uncertain dangers that are inherent to any clinical trial.

The haphazard intentional sampling method requires the formulation of Mixed-Integer Programming optimization problems and the use of numerical optimization software. The haphazard method requires the empirical calibration of an auxiliary parameter that regulates the amount of noise added to the deterministic loss function modeling the unbalance between sample groups. This auxiliary parameter has to be large enough to achieve good decoupling (what corresponds to the haphazard character of the method) and, at the same time, small enough not to disrupt the loss function goal of achieving well-balanced groups (which corresponds to the intentional character of the method). In Section 2, we presented a practical procedure to properly calibrate the auxiliary parameter.

## 5. Final Remarks

This article presented the haphazard intentional sampling method and compared it with the rerandomization method and with the standard randomization method in the context of epidemiological research and medical trials. The first case study concerns a simpler two-group allocation problem, while the second case study extends the method to multiple-group allocation problems. The haphazard method consistently outperformed the rerandomization method which, in turn, consistently outperformed the standard randomization method.

The aforementioned results motivate some topics for further research. In recent years, the performance of Mixed-Integer Quadratic Programming solvers has improved considerably. Hence, in subsequent articles, we shall explore the viability of direct use of the Mahalanobis loss function without recourse to the surrogate linear hybrid loss function. Moreover, we shall conduct a detailed comparative power analysis between all methods at hand and their variations. We hope that the development of more powerful reliable and robust sampling methods will give a significant contribution for handling the COVID-19 pandemics currently afflicting Brazil and the world.

Additionally, we also would like to explore the application of the means and methods developed in this paper to other application areas, such as environmental sciences [25] and data mining [26].

## Figures and Tables

**Table 1:** Parameters $\lambda^{*}$ and maximum CPU time for MILP solver by number of sectors.

Sectors	$\lambda^{*}$	Time (s)
<50	0.1	5
50–4000	0.01	30
>4000	0.001	120

**Table 2:** Root mean square error (RMSE) and standard deviation (SD); red: best result; blue: intermediate result; black: worst result.

City	Haphazard		Rerandomization		Pure Randomization	
	RMSE	SD	RMSE	SD	RMSE	SD
São Paulo	1.6558%	1.6516%	2.4683%	2.3900%	4.9930%	4.9899%
Rorainópolis	0.8582%	0.7487%	1.5116%	1.4310%	3.0028%	3.0008%
Rio de Janeiro	1.3864%	1.3310%	1.9441%	1.9394%	4.6324%	4.6216%
Oiapoque	1.3887%	1.3835%	1.7651%	1.7509%	3.2107%	3.2107%
Marília	1.1624%	1.1603%	1.4787%	1.4737%	3.4950%	3.4919%
Iguatu	0.8329%	0.8196%	1.3029%	1.3025%	3.9094%	3.9003%
Cruzeiro do Sul	1.3873%	1.3489%	2.0482%	2.0457%	5.0029%	5.0003%
Corrente	0.7496%	0.7000%	1.0708%	1.0665%	2.8250%	2.8230%
Campos dos Goytacazes	0.9419%	0.9350%	1.8786%	1.8522%	4.4839%	4.4829%
Brasília	1.7978%	1.3434%	1.5739%	1.5299%	3.9608%	3.9539%

**Table 3:** Efficacy rates for each vaccine [24].

Vaccine	Efficacy (%)
CORONAVAC/SINOVAC (control)	50.4
ASTRAZENECA/OXFORD	70.4
MODERNA	94.5
PFIZER/BIONTECH	95

**Table 4:** Root mean square error (RMSE) and standard deviation (SD); red: best result; blue: intermediate result; black: worst result.

Group	Haphazard		Rerandomization		Pure Randomization	
	RMSE	SD	RMSE	SD	RMSE	SD
1—Coronavac (Sinovac)	0.867%	0.859%	1.881%	1.880%	2.872%	2.872%
2—Pfizer/Biontech	0.092%	0.091%	0.182%	0.181%	0.260%	0.260%
3—AstraZeneca/Oxford	0.499%	0.499%	1.133%	1.130%	1.696%	1.696%
4—Moderna	0.102%	0.102%	0.200%	0.198%	0.311%	0.311%

## Data Availability

Raw data are available at: http://www.epicovid19brasil.org/?page_id=472 (accessed on 4 April 2021). Computer code is available at: https://github.com/marcelolauretto/Haphazard_MaxEnt2021 (accessed on 4 November 2021).

## Abbreviations

The following abbreviations are used in this manuscript:

IBGE	Instituto Brasileiro de Geografia e Estatística
	(Brazilian Institute of Geography and Statistics)
IBOPE	Instituto Brasileiro de Opinião Pública e Estatística
	(Brazilian Institute of Public Opinion and Statistics)
MILP	Mixed-Integer Linear Programming
MIQP	Mixed-Integer Quadratic Programming
RMSE	Root mean square error
SD	Standard deviation
